# Carbon and nitrogen stoichiometry across plant–soil system accounts for the degradation of multi-year alfalfa grassland

**DOI:** 10.3389/fpls.2024.1400261

**Published:** 2024-10-18

**Authors:** Wei Wang, Tao Tian, Meng-Ying Li, Bao-Zhong Wang, Fu-Jian Mei, Ji-Yuan Li, Ning Wang, Yu-Miao Yang, Qiang Zhang, Hong-Yan Tao, Li Zhu, You-Cai Xiong

**Affiliations:** ^1^ Key Laboratory of Soil Environment and Nutrient Resources in Shanxi Province, Shanxi Agricultural University, Taiyuan, China; ^2^ State Key Laboratory of Herbage Improvement and Grassland Agro-ecosystems, College of Ecology, Lanzhou University, Lanzhou, China; ^3^ College of Biology and Agricultural Resources, Huanggang Normal University, Huanggang, China

**Keywords:** alfalfa field, soil aggregates, carbon and nitrogen stoichiometry, degradation, semiarid region

## Abstract

Alfalfa (*Medicago sativa* L.) grassland is prone to degradation following multi-year maintenance. Yet, its mechanism regarding the stoichiometry of carbon (C) and nitrogen (N) across plant–soil system is still unclear. To address this issue, the method of space-for-time sampling was employed to investigate alfalfa grasslands with five planting years (5-, 8-, 10-, 15-, and 20-year periods) in the semiarid Loess Plateau. The results showed that the alfalfa above- and underground biomass decreased steadily decrease after the fifth to eighth years, showing a degradation tendency with the extension of planting duration. The mean weight diameter of aggregate registered an increase with planting years. However, the C and N stocks decreased with planting years in five soil aggregate fractions. Specifically, they were the highest in the fifth year and then started to gradually decrease along the 8th, 10th, 15th, and 20th year. Redundancy and correlation analysis confirmed that the C and N stocks of soil aggregates were closely positively associated with those of plant. Overall, the highest stability of soil physical structure was found during the period from the fifth to eighth year, and, afterward, the stability declined. In conclusion, alfalfa plantation improved soil structure stability but aggravated soil C and N stocks, and biomass and soil aggregate indicators accounted for alfalfa field degradation after a certain year of plantation.

## Highlights

- There exists a steady degradation with planting years in perennial alfalfa field.
- Field productivity and soil aggregate quality declined after 5- to 8-year planting.
- C and N stocks in aggregate fractions were the highest at the fifth year and then declined.
- C and N stocks of soil aggregates were positively linked with that of alfalfa (*p* < 0.05).
- Steady deterioration of C and N stocks from soil to plant explains system degradation.

## 1 Introduction

The Loess Plateau is one of the most seriously degraded soil regions, which is located in northern China (Li et al., 2021; Yuan et al., 2024). Soil degradation is related to the climatic, environmental, and geological features of this region (Gong et al., 2020); it is also strongly tied to unreasonable land-use management (Fan et al., 2016). To restrain soil degradation, alfalfa (*Medicago sativa* L.) was extensively planted until the 1960s in the Loess Plateau due to the characteristics of the nutrient-rich forage crop with high biological yield potential, high resistance ability, and wide adaptability in extreme environments (Zhang X. L. et al., 2021; Song et al., 2024). Alfalfa has the potential to enhance nitrogen (N) input into soil ecosystems while promoting soil carbon (C) and N accumulation (Vitousek et al., 2013). However, alfalfa forage biomass is gradually reduced with the duration of planting years, and continuous planting for many years will degrade the alfalfa biomass and soil quality (Gu et al., 2018; Fang et al., 2021; Qi et al., 2023). Therefore, it is essential to study the optimum cultivation period and the degradation mechanism of alfalfa grasslands.

Ecological stoichiometry was used to explore the degradation mechanism for plant responses to environmental change (Austin and Vitousek, 2012). The ecological stoichiometry of C and N across ecosystem components can provide a new strategy for elucidating the nutrient cycle process (Du and Gao, 2021; Tang et al., 2022). Carbon is an essential energy source for above- and underground ecosystem biogeochemical processes (Wang et al., 2021b), whereas N is an essential plant nutrient as a key limiting factor determining primary production in ecosystems (Wang et al., 2021a). The C and N stoichiometry of plants and their interaction with the soil significantly affect ecosystem functions (Yang et al., 2019; Xu et al., 2024). Soil C and N availability affects plant nutrient absorption and assimilation (Zhang et al., 2019); In turn, plant litter and root exudates will provide substrates to enhance soil C and N cycling processes (Zhou et al., 2019; Dan et al., 2023). Overall, the variation in C and N cycles in ecosystems can change the stoichiometry of plants and soils (Wang et al., 2021a). C and N stoichiometric flexibility might affect terrestrial ecosystem biogeochemical cycling, a changing the ecosystem productivity and terrestrial degradation (Sardans et al., 2017; Li et al., 2023). Therefore, it is necessary to determine the C and N stoichiometry in plants and soil to explore the degradation mechanism of perennial alfalfa grasslands.

Soil aggregates are composed of granular or small clumped structures, and soil aggregate stability is a key index for characterizing soil degradation (Zhu et al., 2018; Tan et al., 2024). Soil aggregates are affected by many factors, such as the soil physical and chemical properties, soil microbes, plant root distribution, and artificial cultivation (Bodner et al., 2014; Shen et al., 2024). Based on soil size, aggregates are classified into three parts: macroaggregates (>0.25 mm), microaggregates (0.053–0.25 mm), and the silt-clay fraction (<0.053 mm) (Lu et al., 2021). The soil aggregate size distribution often influences soil aeration, corrosion resistance, and water permeability (Bissonnais and Arrouays, 1997). Soil aggregation is a process driven the biotic and abiotic factors that plant-derived organic matter input, microorganisms, and soil conditions combination (Liu et al., 2023). Increasing plant cover to prevent the surface erosion and to protect soil physical disturbance promotes the plant-derived organic matter input and contributes soil aggregation formation (Wang et al., 2022). In addition, more plant residue input also promotes the microbial grower, regulate microbial activity, and microbial-derived C and ultimately affects the formation of soil aggregate (Zhao et al., 2024). Higher C concentrations and mineralization rates are often found to be associated with macroaggregates (Wang et al., 2018; Araujo et al., 2024). This is because macroaggregates are easily disintegrated and broken by external interference, whereas microaggregates may be more physically protected and are, therefore, more biochemically recalcitrant to C mineralization (Kubar et al., 2021). Previous studies have investigated the soil aggregates in various land-use types, including forests, cultivated lands, grazing lands, and woodlands. In fact, most of above investigations on soil aggregates are mainly aimed at the stability and C and N characteristics (Wang et al., 2018; Bhatt et al., 2023), and few studies have focused on the relationship between alfalfa aggregates and plant C and N stoichiometry. Therefore, knowledge of C and N stoichiometry is crucial for understanding the biochemical mechanisms of plant and soil aggregates.

Plant–soil interactions via both positive and negative feedbacks can constrain or promote plants development in the novel environment, and plant–soil interaction is strongly influenced by the rhizosphere microbiota (Lustenhouwer et al., 2024). Plant–soil interaction plays an important role in global C and N biogeochemical cycles, and previous study found that rhizobacteria symbiotic with legumes can drive positive effect, and promote nutrient uptake and plant growth (Yahui et al., 2023). However, there is still controversy about how multi-year alfalfa planting affects the C and N in soil aggregates and plant organs in the semiarid regions. We hypothesized that the dynamics of C and N sequestration might be key indicators to explain soil degradation following continuous alfalfa plantation. To clarify this issue, the method of space for time was employed to investigate 5-year succession of alfalfa grasslands in the semiarid region. The purposes of this study were 1) to determine the changes in the production of alfalfa with planting years, 2) to identify the stocks dynamics of C and N in soil aggregate distribution with planting years, 3) to reveal the changes C and N across plant–soil system in the multi-year alfalfa grassland, and 4) to explore the degradation mechanisms of perennial alfalfa. The findings will help formulate an appropriate management strategy for ecological restoration and sustainable soil development.

## 2 Materials and methods

### 2.1 Description of the study site

**Figure 1:**
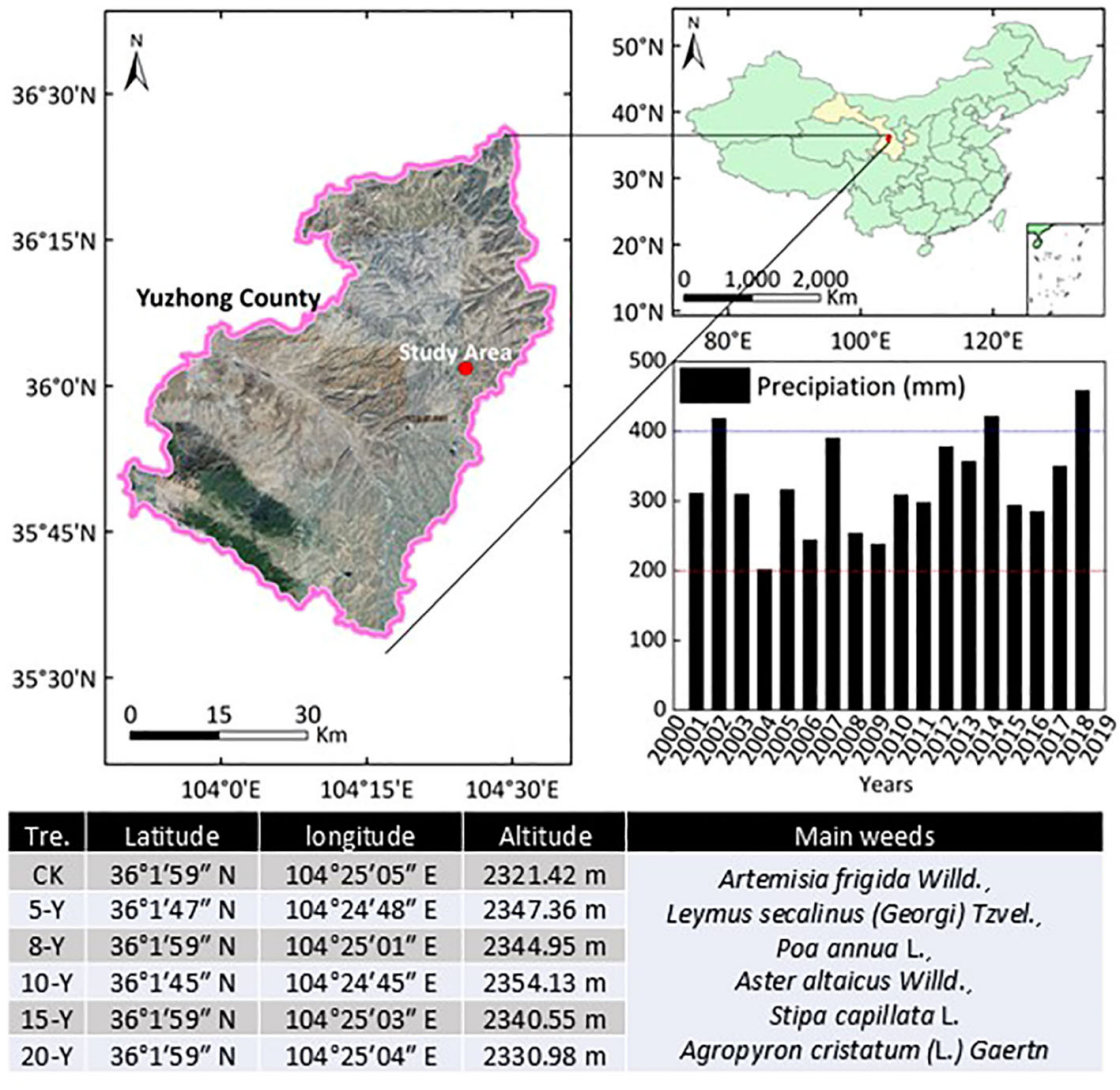
Location of study area and its precipitation and biodiversity background. CK, abandoned farmland. 5-Y, 8-Y, 10-Y, 15-Y, and 20-Y planting indicate the variations across 5, 8, 10, 15, and 20 years, respectively.

The study area is located in Yuzhong County, Gansu Province, China (36°02′N, 104°25′E, 2,400 m) (**Figure 1**). The average annual temperature is 6.5°C, and the monthly average maximum and minimum temperatures are 19.0°C and −8.0°C, respectively. The mean annual precipitation from 2000 to 2018 was 323.5 mm, the maximum year was 2018 (458.8 mm), and the minimum precipitation occurred in 2004 (202.0 mm). The average annual water evaporation is approximately 1,450 mm. The local soil type is Calcic Kastanozems (Siltic) or rusty dark loess soil with a pH of 8.2 (Guo et al., 2010). The rainfall data were derived from the perennial positioning automatic meteorological recording instrument (**Figure 1**).

### 2.2 Experimental design

We selected five alfalfa grasslands in October 2018 and formed a time series of 5, 8, 10, 15, and 20 years, which represented successional series with similar topographic conditions. Where fields planted with alfalfa were recorded in 2013, 2010, 2008, 2003, and 1998, respectively. Abandoned farmland was chosen as the control group (CK; abandoning 15 years). The aspect and slope of each selected alfalfa field remained relatively consistent (**Figure 1**). In all the selected plots, the alfalfa variety *Algonquin* was planted, each plot was sown with alfalfa seeds of 35 kg ha^−1^, the depth was approximately 2 cm, and each row was almost 20 cm. There was no irrigation throughout the growing season. Fertilization was only applied in the first year of planting (N, 185.6 kg ha^−1^; P, 48 kg ha^−1^), and no fertilization was conducted afterward. There was no grazing treatment for the alfalfa grassland, which was harvested in June and October of each year (weeds were also mowed at the same time). The control group had no fertilization and mowing treatment. The only difference in the selected alfalfa grassland was in planting years. In the alfalfa grassland, weed biomass increased with prolonged plantation years. The dominant vegetation species are *Artemisia frigida Willd*., *Leymus secalinus (Georgi) Tzvel.*, *Poa annua* L., *Aster altaicus Willd*., *Stipa capillata* L., and *Agropyron cristatum* L. *Gaertn*. The weeds were periodically cleared by hand when needed to avoid interspecific disturbance.

### 2.3 Sampling

In October 2018, 15 plots of 1 m × 1 m of each field were randomly selected and marked to measure the biomass of each field. Soil samples of 0-cm to 20-cm depth were randomly collected from the field using a soil auger (diameter of 5 cm) with five replicates for each planting period, and each alfalfa field was mixed in three samples. Before air-drying, the soil was sieved through 2.0-mm screens for available nutrient analyses, and, after drying, it was sieved through 0.15-mm screens for total nutrient analyses. The soil aggregate sample was collected with a hard Polyvinyl Chloride (PVC) pipe with a diameter of 10 cm and a height of 20 cm. After sampling, the aggregate sample was divided into small clods a diameter of 1 cm for air-drying.

### 2.4 Physical and chemical analysis

Soil bulk density (SBD) was determined using cutting ring (0–20 cm). Soil water storage (SWC) was measured gravimetrically (0–20 cm, 20–40 cm, 40–60 cm, 60–80 cm, and 80–100 cm). The soil aggregate-size fraction was measured using a dry- and wet-sieving method (Su et al., 2009). For the dry sieving method, we used 100 g of air-dried soil samples in a stainless-steel vibrating sieve divided into five sizes: >2 mm, 2–1 mm, 1–0.5 mm, 0.5–0.25 mm, and <0.25 mm. The wet sieving method used 50 g of air-dried soil, which was weighed according to dry sieving and moistened for 10 min with distilled water. The apparatus specifications were an oscillation of 10 min at a frequency of 30 cycle min^−1^. Aggregates retained in the sieve were air dried at 65°C, weighed, and stored for C and N content measurements. The soil aggregate distribution is calculated with Equation 1. The mean weight diameter (MWD) and percentage of aggregate destruction (PAD) are calculated using Equations 2 and 3, respectively.


(1)        AD=ADi/TO×100%

(2) $$\text{MWD}=\left[\sum_{\text{i}=1}^{\text{n}}\left(\bar{\text{Xi}}\text{ ADi}\right)\right]/\sum_{\text{i}=1}^{\text{n}}\text{ADi}$$

(3)  PAD= (ADd−ADw)/ADd

where AD is the aggregate distribution of each fraction (%), ADi is the i size aggregate weight (g), and TO is the total weight of the soil (g). Xi is the mean diameter of the aggregate, and ADd and ADw are the proportions of dry and water stable aggregates (>0.25 mm), respectively.

The SOC content and C of aggregate fractions are determined by Puget et al. (2000). The soil total nitrogen (TN) content and TN of the aggregate fractions were measured by Bremner (1997). The above- and underground biomass of alfalfa and weeds (soil depth up to 40 cm) were collected from a 1 m × 1 m area of each plot (collected twice in late June and early October). The above- and underground biomass were dried at 105°C for 30 min and then at 65°C to constant weight (Wilson et al., 2009). The C and N contents of alfalfa plants were measured following Yu et al. (2021); aggregate C and N stocks were measured according to Equation 4 (Zhang L. Q.  et al., 2015).


C(N)stock=ADi ×M×SOCi(TNi)×0.001 

(4)   M=SBD×H×0.01×10000

where C and N stocks are stocks of soil carbon and nitrogen [Mg ha^−1^; where M is soil quality per unit area (Mg ha^−1^)], SOCi and TNi are different size aggregates of soil carbon and nitrogen content (g kg^−1^), SBD is the soil bulk density (g cm^−3^), and H is 20 cm.

### 2.5 Statistical analysis

All statistical data were tested for normality and homogeneity of variance before further analysis. One-way ANOVA was used to compare the differences among the six treatments. The difference was tested by Tukey’s honestly significant difference (HSD) to check the statistical significance of the treatments between different planting years. Mean comparisons were performed using the least significant difference at a probability level of 0.05. Redundancy analysis (RDA) and correlation analysis were used to evaluate the relationship between aggregate C and N stocks, plant biomass, and the C and N concentrations of alfalfa plants. RDA was plotted using Canoco 5.0. Graphs were prepared using ArcGis 10.0 and Origin 2021.

## 3 Results

### 3.1 Dynamics of aboveground and belowground biomass with planting years

Above- and underground biomass varied significantly among alfalfa cultivation years (**Table 1**). Throughout various planting for alfalfa, a significant change in above- and underground biomass was occurred. In the aboveground biomass, alfalfa yields reached the highest after approximately 5 years, whereas those of the remaining years declined approximately 20 years after alfalfa planting. Conversely, underground biomass was at a maximum value after alfalfa planting for 8 years but then decreased with the duration years. Therefore, the above- and underground biomass of alfalfa grassland declined with the number of years of planting.

**Table 1:** Variations of above- and belowground biomass and the C and N stocks in alfalfa along varying planting durations.

Years	Aboveground				Underground			
	Biomass (kg ha^−1^)	C content (g kg^−1^)	N content (g kg^−1^)	C-N ratio	Biomass (kg ha^−1^)	C content (g kg^−1^)	N content (g kg^−1^)	C-N ratio
CK	2,349.3 ± 23.1e	522.8 ± 1.2a	6.42 ± 0.0e	81.5 ± 0.0a	1,866.3 ± 11.5f	413.3 ± 1.1f	4.11 ± 0.1e	100.9 ± 2.6a
5-Y	3,985.6 ± 11.5a	506.1 ± 1.1b	16.4 ± 0.1bc	30.8 ± 0.1b	3,906.3 ± 23.1e	588.4 ± 1.1a	9.83 ± 0.1c	59.8 ± 0.6b
8-Y	3,788.3 ± 11.5b	475.2 ± 1.2c	16.9 ± 0.2a	28.5 ± 0.1c	5,757.3 ± 11.5a	520.6 ± 1.2b	9.26 ± 0.2d	56.2 ± 0.8c
10-Y	3,516.0 ± 11.4c	463.5 ± 1.2d	16.7 ± 0.1ab	27.7 ± 0.1d	5,542.6 ± 11.5b	513.9 ± 1.1c	11.8 ± 0.1b	43.8 ± 0.3d
15-Y	2,743.9 ± 11.7d	451.2 ± 0.6e	16.3 ± 0.1c	27.7 ± 0.2d	5,259.3 ± 11.5c	456.0 ± 1.1d	12.2 ± 0.2a	37.6 ± 0.3e
20-Y	1,925.4 ± 23.1f	432.1 ± 1.2f	15.8 ± 0.2d	27.3 ± 0.1e	4,856.4 ± 5.8d	440.2 ± 1.2e	12.1 ± 0.2a	36.4 ± 0.2e

Different lowercase letters indicate significant differences among the same parameter at *p* < 0.05 according to Tukey HSD tests. CK, abandoned farmland. 5-Y, 8-Y, 10-Y, 15-Y, and 20-Y represent planting variations for 5, 8, 10, 15, and 20 years, respectively.

### 3.2 Dynamics of the organic carbon and total nitrogen concentrations of plants with planting years

The C and N contents and C-N ratio of alfalfa varied with planting years (**Table 1**). The C contents of alfalfa above- and underground decreased after continuous planting 5 years. The N content of the aboveground also showed significant change; however, the N content of underground significantly increased with planting years. Similarly, the C-N ratio of alfalfa above- and underground also declined under cultivated 5 years.

### 3.3 The responses of soil water content and soil bulk density to planting years

**Figure 2:**
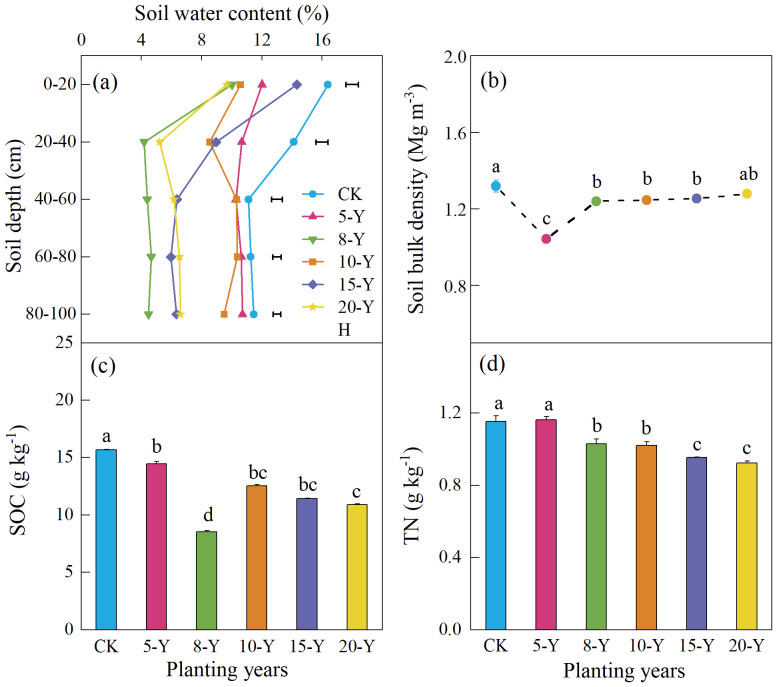
Dynamics of soil water content (SWC; 0–100 cm) **(A)**, soil bulk density **(B)**, SOC content **(C)**, and TN content **(D)** at 0-cm to 20-cm soil depth in response to different durations of alfalfa planting. Different letters indicate significant differences in planting duration (*p* < 0.05). The values are mean + SE (error bar). CK, abandoned farmland. 5-Y, 8-Y, 10-Y, 15-Ym and 20-Y represent planting variations for 5, 8, 10, 15, and 20 years, respectively.

As the depth of the soil layer increases, the SWC of the 0-cm to 100-cm soil layer decreases significantly, and the soil moisture content of alfalfa grassland is significantly lower than that of the control (**Figure 2A**). At different planting times, the SWC was the highest after 10 years of planting and the lowest after 8 years of planting. The SBD of alfalfa grassland ranged from 1.04 g cm^−3^ to 1.28 g cm^−3^, and there was a significantly variable trend in 5 years, and there is less change after cultivated 8 to 20 years (**Figure 2B**). The change in SBD after 8, 15, and 20 years of planting was significantly higher than that after 5 years of planting, followed by 10 years of planting.

### 3.4 The dynamics of soil organic carbon and nitrogen contents under different planting years

The soil organic carbon (SOC) content of artificial alfalfa grassland at 0-cm to 20-cm soil depth showed a V-shaped trend with increasing alfalfa planting duration. The highest value of planting for 5 years was 14.4 g kg^−1^, and the lowest value of planting for 8 years was 8.54 g kg^−1^ (**Figure 2C**). The soil TN content was between 0.89 g kg^−1^ and 1.02 g kg^−1^, and the planting years had a significant effect on soil TN content, which showed a decreasing trend with increasing planting years (**Figure 2D**).

### 3.5 The dynamics of soil aggregate distribution with planting years

In general, the distribution of soil aggregates in size ranged considerably among various years of cultivation (**Table 2**). The proportion of aggregates with sizes > 2 mm and < 0.25 mm is significantly higher than that of aggregates with other sizes. After 8 years of planting, the proportion of aggregates > 2-mm size was higher than that for other planting durations, the proportions of aggregates of 1.0–2.0 mm, 0.5–1.0 mm, and 0.25–0.5 mm after 5 years of planting were higher than those for other planting durations (except for the 1.0–2.0 mm). The proportion of <0.25-mm aggregates after 20 years of planting was higher than that for other periods of cultivation.

**Table 2:** Responses of soil mechanical and water-stable aggregate compositions to varying planting years in alfalfa field.

Years	Composition of soil mechanical stable aggregate (%)					R_0.25_ (%)	MWD (mm)	
	< 0.25 mm	0.25–0.5 mm	0.5–1.0 mm	1.0–2.0 mm	> 2.0 mm			
CK	45.4 ± 1.14BCa	3.96 ± 0.38Ab	3.86 ± 0.27Ab	5.05 ± 0.24Ab	41.7 ± 0.51Ca	54.6 ± 1.14BC	1.01 ± 0.01C	
5-Y	37.1 ± 2.04Db	3.70 ± 0.04Ac	4.86 ± 0.42Ac	3.88 ± 0.22Bc	50.5 ± 2.30Aa	62.9 ± 2.04A	1.16 ± 0.04A	
8-Y	40.4 ± 0.99CDb	3.49 ± 0.14ABc	2.02 ± 0.25Bc	2.41 ± 0.25Cc	51.7 ± 0.93Aa	59.6 ± 0.99AB	1.15 ± 0.02AB	
10-Y	46.8 ± 1.06Ba	2.72 ± 0.04BCb	1.52 ± 0.21Bb	1.67 ± 0.25CDb	47.3 ± 1.05ABa	53.2 ± 1.06C	1.05 ± 0.02BC	
15-Y	53.0 ± 0.55Aa	1.84 ± 0.25CDc	1.46 ± 0.12Bc	1.63 ± 0.14CDc	42.1 ± 0.23BCb	47.0 ± 0.55D	0.95 ± 0.01CD	
20-Y	56.3 ± 0.73Aa	1.50 ± 0.04Dc	1.21 ± 0.03Bc	1.32 ± 0.03Dc	39.6 ± 0.72Cb	43.7 ± 0.73D	0.90 ± 0.01D	
Years	Composition of soil water stable aggregate (%)					R_0.25_ (%)	MWD (mm)	PAD (%)
	< 0.25 mm	0.25–0.5 mm	0.5–1.0 mm	1.0–2.0 mm	> 2.0 mm			
CK	81.0 ± 0.94CDa	3.36 ± 0.32Ac	2.61 ± 0.19Bc	1.40 ± 0.16Ac	11.6 ± 0.50Bb	19.0 ± 0.94B	0.39 ± 0.01B	65.3 ± 1.27A
5-Y	76.3 ± 0.69Ea	3.14 ± 0.04Ac	3.80 ± 0.03Ac	1.44 ± 0.08Ac	15.3 ± 0.58Ab	23.7 ± 0.69A	0.46 ± 0.01A	62.2 ± 2.30A
8-Y	78.2 ± 0.85DEa	2.63 ± 0.22ABc	2.38 ± 0.13Bc	1.45 ± 0.29Ac	15.3 ± 0.35Ab	22.1 ± 0.57A	0.45 ± 0.01A	62.9 ± 1.40A
10-Y	82.5 ± 0.49BCa	2.30 ± 0.03BCc	1.74 ± 0.10Cc	1.33 ± 0.11Ac	12.1 ± 0.48Bb	17.5 ± 0.49BC	0.39 ± 0.01B	67.1 ± 0.29A
15-Y	85.0 ± 0.65ABa	1.61 ± 0.07Cc	1.31 ± 0.05CDc	0.86 ± 0.03Ac	11.2 ± 0.58Bb	15.0 ± 0.65CD	0.36 ± 0.01B	68.1 ± 1.32A
20-Y	85.8 ± 0.36Aa	1.55 ± 0.01Cc	1.16 ± 0.03Dc	0.83 ± 0.03Ac	10.7 ± 0.32Bb	14.2 ± 0.36D	0.35 ± 0.01B	67.4 ± 1.27A

Different capital letters indicate significant differences among different planting durations in the same-sized aggregate at the 0.05 level, and different small letters indicate significant differences among different-sized aggregates in the same planting durations at the 0.05 level. The values are mean + SE. R_0.25_, aggregates of diameter >0.25 mm; MWD, mean weight diameter; PAD, percentage of aggregate destruction; CK, abandoned farmland. 5-Y, 8-Y, 10-Y, 15-Y, and 20-Y represent planting variations for 5, 8, 10, 15, and 20 years, respectively.

In addition, both the mechanical and water-stable aggregates, soil aggregate, R_0.25_ and MWD showed a declining trend with increase planting years. The mechanical and water-stable aggregates R_0.25_ ranged from 62.9% to 43.7% and from 23.7% to 14.2% accordingly. The mechanical and water-stable aggregates MWD change from 1.16 mm to 0.90 mm and from 0.46 mm to 0.35 mm, respectively. In contrast, PAD presented an increasing trend and ranged from 62.2% for planting for 5 years to 67.4% for planting for 20 years.

### 3.6 The soil organic carbon and total nitrogen contents of different aggregates in response to planting years

**Figure 3:**
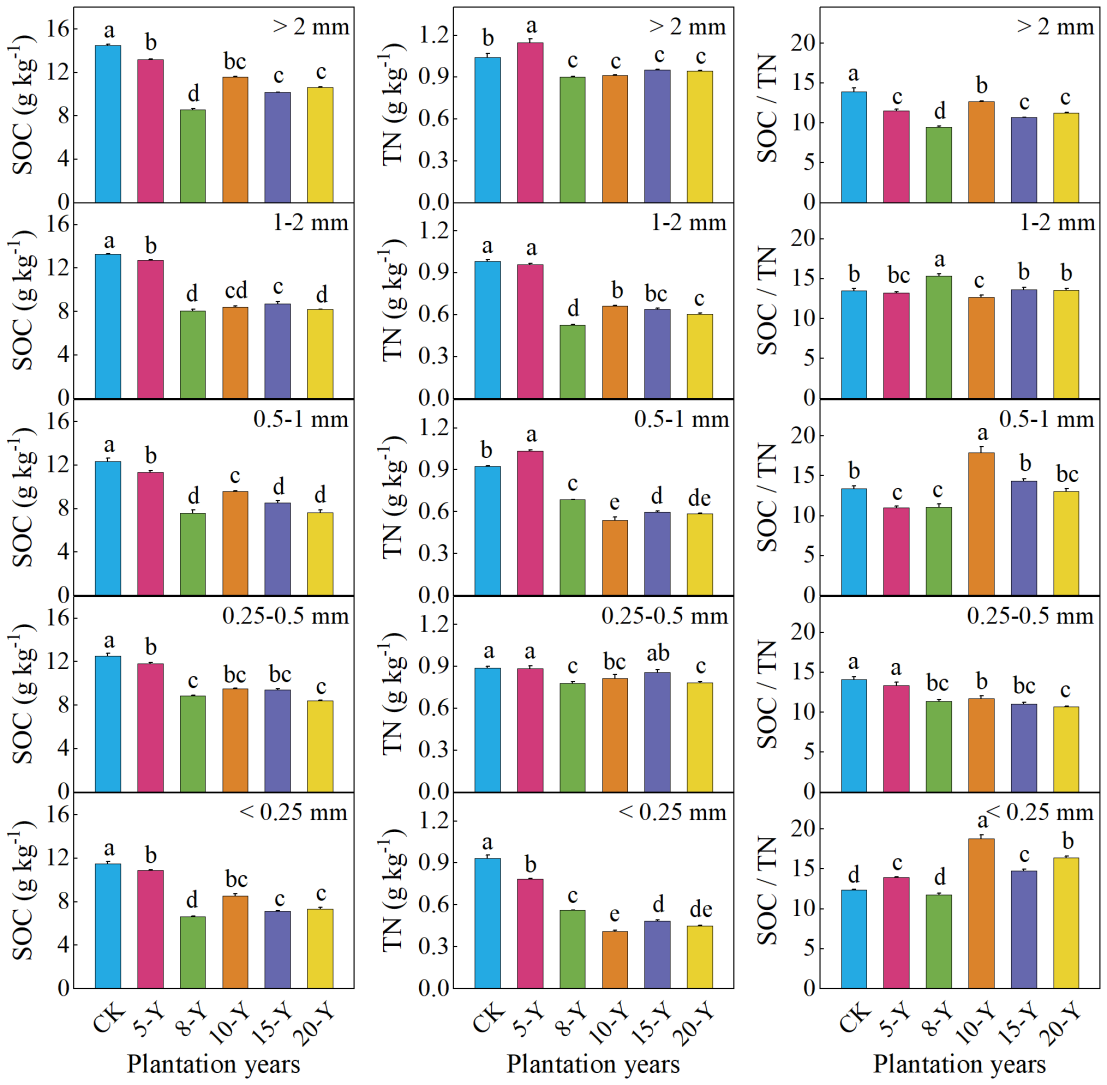
SOC, TN contents, and SOC/TN of different-sized aggregates at 0-cm to 20-cm soil depth in alfalfa plantations with different planting durations. Different letters indicate significant differences among different planting durations for the same-sized aggregate at the 0.05 level. The values are mean + SE (error bar). CK, abandoned farmland. 5-Y, 8-Y, 10-Y, 15-Y, and 20-Y represent planting variations for 5, 8, 10, 15, and 20 years, respectively.

The soil organic carbon (SOC), TN, and C-N ratios in different aggregates of alfalfa grassland showed a variable trend in planting years. The SOC and TN contents of each size were the highest at 5 years of planting, but the lowest was at 8 years of planting. The C-N ratio in all five sizes showed that the highest value was in the 10-year plantation, and the lowest was in the 5-year plantation (except for the 1–2 mm) (**Figure 3**).

In addition, the SOC and TN contents and C-N ratio of different size aggregates in the same planting years also showed a significant difference (**Figure 3**). In general, the SOC and TN contents of macroaggregates in the alfalfa grassland were higher than those in other particle sizes, and the < 0.25-mm particle size was the lowest. In the alfalfa grassland field, the C-N ratio of >2 mm, 0.25–0.5 mm, and <0.25 mm in 8 years was the lowest than that in the other years. After planting for 8 years, the ratios of the five size aggregates increased in all five sizes.

### 3.7 The dynamics of carbon and nitrogen stocks with planting years

**Figure 4:**
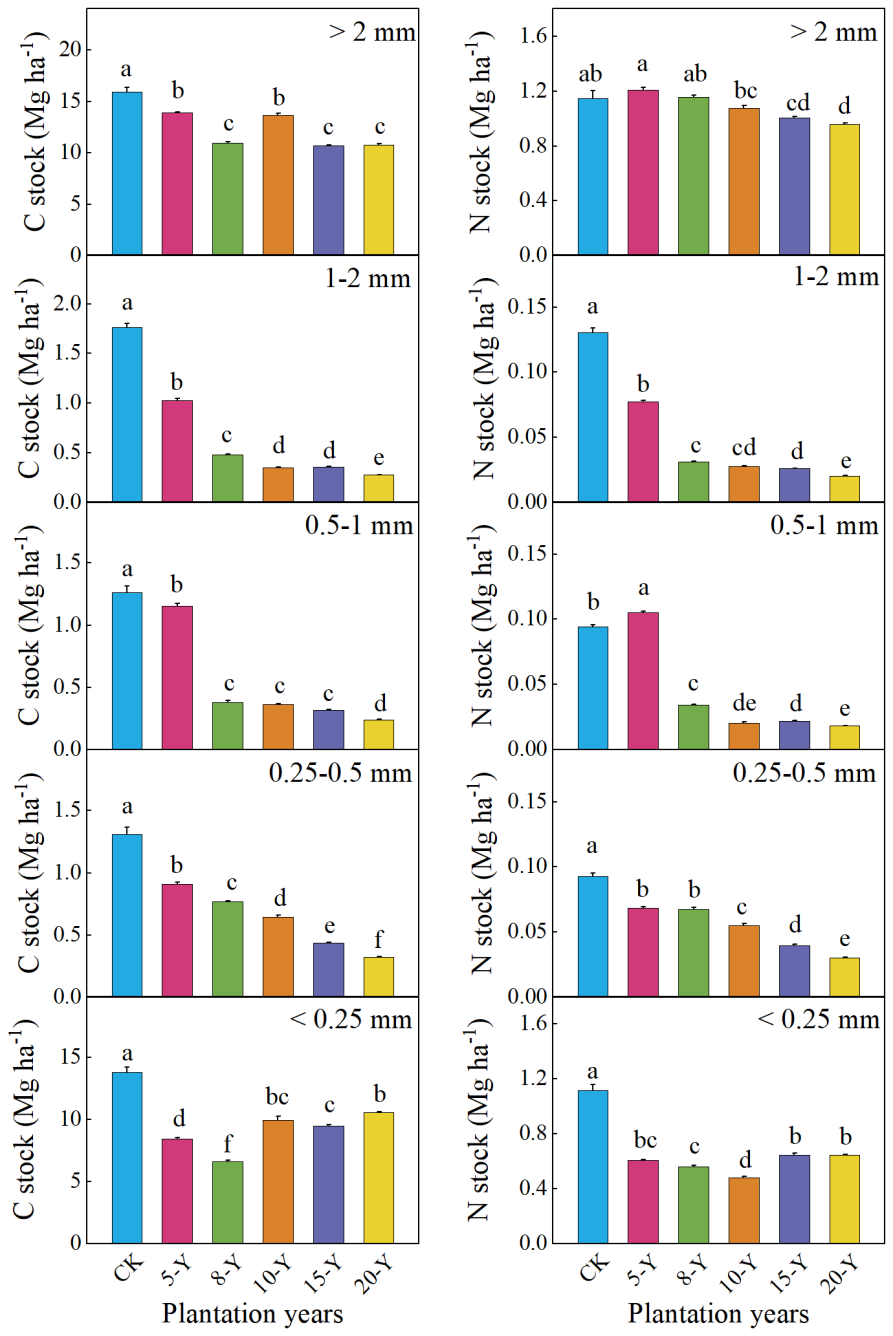
C and N stocks of different-sized aggregates at 0- to 20-cm soil depth in alfalfa plantations with different planting durations. Different letters indicate significant differences among different planting durations for the same-sized aggregate at the 0.05 level. The values are mean + SE (error bar). CK, abandoned farmland. 5-Y, 8-Y, 10-Y, 15-Y, and 20-Y represent planting variations for 5, 8, 10, 15, and 20 years, respectively.

The variation in the C and N stocks of the alfalfa succession series with different planting years is shown in **Figure 4**. In general, the C and N stocks of aggregates > 2 mm were the highest in each planting year (except for <0.25 mm). Among different planting years, the stock of C after 5 years of planting was the highest, and, after 8 years, it was declined. Similarly, in each planting year, the N stocks of aggregates > 2 mm were the largest. The N stocks after 5 years were the highest among the five planting years. Overall, the C and N stocks slightly decreased with prolonged planting.

### 3.8 Relationships of aggregate carbon and nitrogen stocks with alfalfa plants

**Figure 5:**
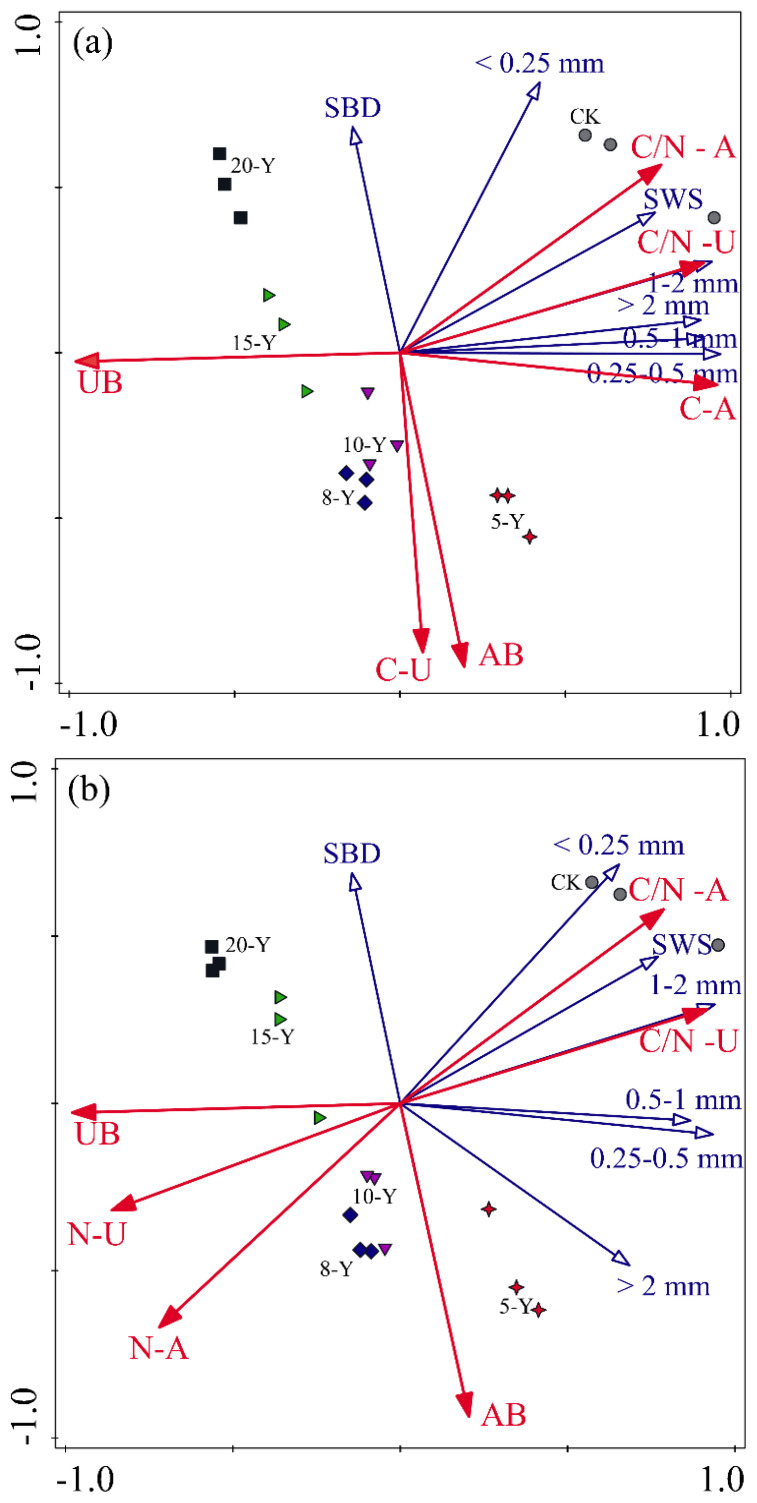
RDA biplot based on biomass and C and N concentrations of alfalfa plant variables in all treatments under different size aggregates C **(A)** and N **(B)** stocks. CK, abandoned farmland. 5-Y, 8-Y, 10-Y, 15-Y, and 20-Y represent planting variations for 5, 8, 10, 15, and 20 years, respectively, AB, aboveground biomass; UB, underground biomass; C-A, carbon concentration of alfalfa aboveground plant; C-U, carbon concentration of alfalfa underground plant; N-A, nitrogen concentration of alfalfa aboveground plant; N-U, carbon concentration of alfalfa underground plant; C/N-A, carbon-nitrogen ratio of alfalfa aboveground plant; C/N-U, carbon-nitrogen ratio of alfalfa underground plant; SBD, soil bulk density; SWS, soil water storage.

The RDA showed that the CK treatment was concentrated in the first quadrant, and alfalfa grasslands were distributed in the other three quadrants (**Figure 5**). The first and second axes accounted for 84.63% (a) and 84.62% (b) of the variability explained. Aboveground biomass was negatively correlated with SBD and planting years, whereas underground biomass was positively correlated with planting years. At the same time, we found that aggregate C and N stocks were closely related to the C-N ratio of alfalfa above- and underground plants.

**Figure 6:**
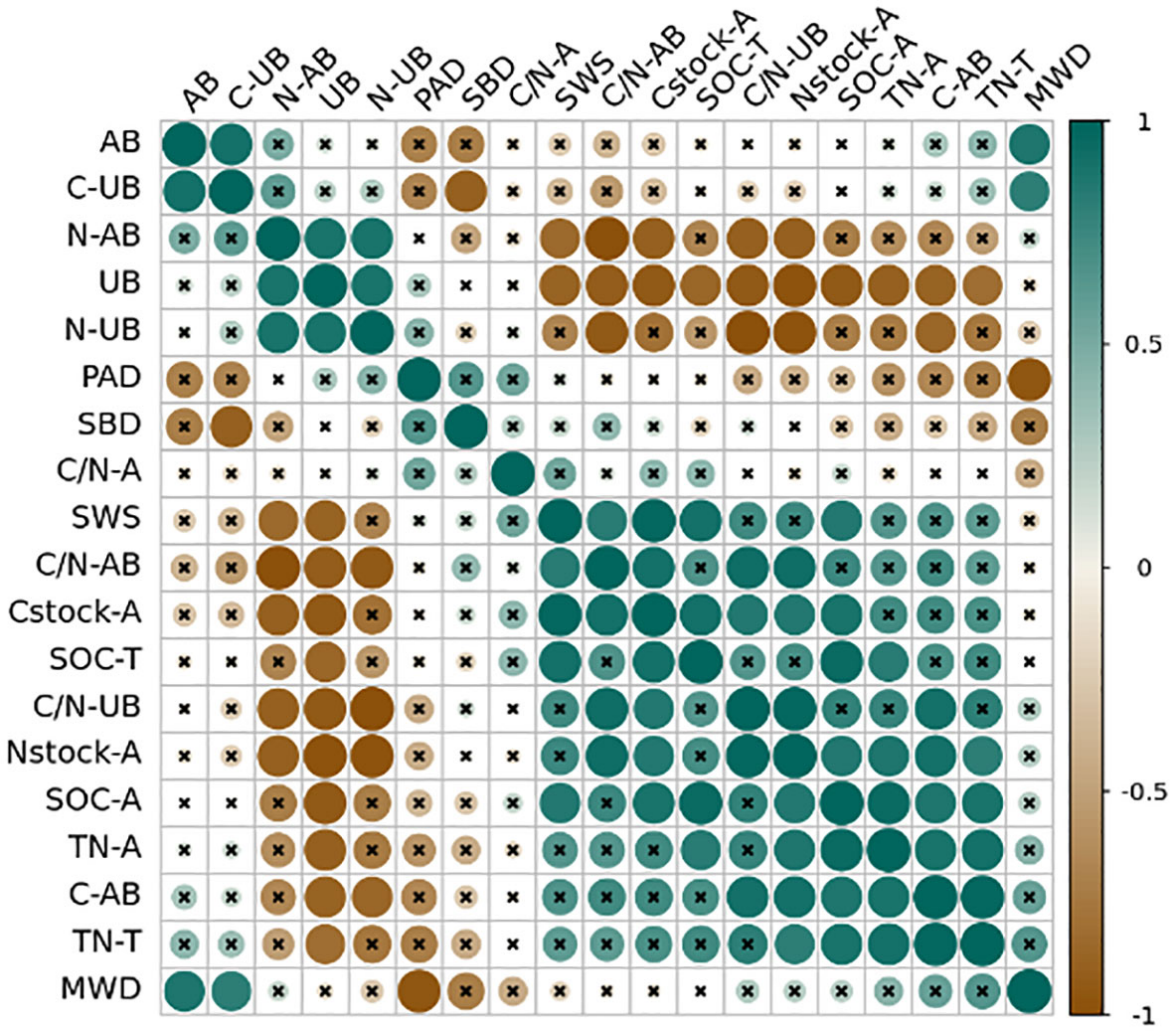
Correlation matrix of the study variables. Color and the size of the circles are proportional to the correlation coefficients between the variables. SWS is soil water storage, SBD is soil bulk density, SOC-T is surface soil organic carbon content of 0-cm to 20-cm depth, TN-T is surface soil total nitrogen content of 0-cm to 20-cm depth, MWD is the mean weight diameter, PAD is the percentage of aggregate destruction, SOC-A is the average SOC content of five size aggregates, TN-A is the average TN content of five size aggregates, Cstock-A is the sum of SOC stock in the five size aggregates, Nstock-A is the sum of TN stock in the five size aggregates, AB is the aboveground biomass, UB is the underground biomass, C-AB is the SOC content of aboveground plant, C-UB is the SOC content of root, N-AB is the TN content of aboveground plant, N-UB is the TN content of root, C/N-AB is the C/N content of aboveground plant, C/N-UB is the C/N content of root. The values in the figure represent correlation coefficients.

The correlation analysis indicated that aggregate MWD was positively correlated with alfalfa aboveground biomass and the above- and underground C content (**Figure 6**). The aggregate C stock was markedly positively correlated with the SWC, the C and N content of aggregates, the C content of alfalfa aboveground, and the C-N ratio of above- and underground. Similarly, the aggregate N stock was markedly positively correlated with the SWC, C and N content of the soil surface and each aggregate size, the aboveground C content of alfalfa, and the aboveground C-N ratio. The C and N stocks of aggregates were negatively correlated with underground biomass and the above- and underground N content. In addition, the aboveground C content of alfalfa was positively correlated with SWC and MWD. The above- and underground N content was negatively correlated with the SWC and aboveground C content.

## 4 Discussion

### 4.1 The dynamic of alfalfa productivity under cultivation years

The productivity of alfalfa is affected by soil fertility, soil physical conditions, and other ecological factors (Feng et al., 2022). Soil moisture will affect the growth of roots, and excessive or limited water supply will change the distribution of crop roots, thereby affecting yields (Jia et al., 2009; Fan and Schutze, 2024). The results of this study showed that the largest aboveground biomass was found after 5 years in a cultivated field (**Table 1**), which is related to the precipitation in previous and subsequent years (**Figure 1**). Water is the main limiting factor restricting vegetation restoration and reconstruction, and it determines the water–soil ecological relationship of grassland on the Loess Plateau (Jiang et al., 2006). Soil moisture is affected by many factors, such as soil characteristics, land-use structure, topography, vegetation types, and weather, resulting in complex dynamic changes (Fan et al., 2016). In this paper, compared to the control, the soil water content of alfalfa with different planting years has a greater decrease (**Figure 2A**), which indicates that the soil water deficit is obvious. Because the local soil is loess soil, it is beneficial for rainwater to penetrate the soil and restore the water content. On the other hand, we found that the root biomass was negatively correlated with SWC (**Figure 6**), which may be because the root system of alfalfa is relatively wide, and the proliferation of the root system in the soil increases the porosity of the soil (Jia et al., 2009; Heinze, 2020). In addition, as the planting time increases, the distribution of root systems becomes wider and deeper, which often leads to more consumption and utilization of shallow soil water (Li and Huang, 2008). Moreover, as the planting time increases, the soil moisture gradually decreases, which is directly related to the root biomass. The accumulation of root biomass requires sufficient soil moisture, which leads to water depletion in the soil profile. In addition, alfalfa productivity was also affected by local rainfall (Zhang X. L. et al., 2021). In our study, we found that planting alfalfa for 5 years had the highest aboveground biomass, which may be related to the increasing rainfall from 2010 to 2013, and, after 2013 years, alfalfa biomass showed a declining trend due to the relative decreasing precipitation (**Figure 1**).

### 4.2 The driving factors of soil aggregate distribution in alfalfa grasslands

Soil aggregates are closely connected with soil physical, chemical, and biological properties (Plaza et al., 2013; Li et al., 2019). In this experiment, the particle size distribution of soil aggregates in different years of alfalfa cultivation and abandoned land showed a “V”-shaped trend, and soil aggregate size distributions of >2 mm and <0.25 mm were dominant (**Table 2**). This can be further explained by the fact that the stability of loess soil is generally low. Therefore, macroaggregates will decompose into microaggregates or smaller soil particles after being immersed in water (Lu et al., 2021). In addition, alfalfa planting for numerous years significantly increased the macroaggregate content in certain years, which is directly related to the proliferation of alfalfa roots. Because the root biomass can be increased by producing thick roots or multiple thin roots (Heinze, 2020), the increase in planting time will lead to more accumulation of root biomass, and this extensively proliferating root system helps to consolidate broad soil particles from smaller ones (Tisdall and Oades, 1982). However, due to the reduced physical and mechanical disturbance, the surface herbs, litter, and root biomass increase in natural-restored grassland, resulting in increased soil organic carbon, which is beneficial for aggregate formation (Xiao et al., 2019). Similar outcomes have been reported by Song et al. (2024) who found that the plant residue accelerates the soil aggregates turnover, promoting the soil macroaggregate formation. In addition, we found that dissolved organic matter that, as a substrate for microorganism, contributes to the increase in microbial activity may contribute to macroaggregate formation and stability (Sonsri and Watanabe, 2023).

### 4.3 The driving factors of plant–soil C and N stoichiometry of alfalfa grasslands

Plant–soil C and N concentrations and their ratios are essential in the restoration process of grasslands, not only for a better understanding of the C and N cycles but also for management practices (Du and Gao, 2021). We evaluated alfalfa above- and underground plant C and N contents and their ratios during the multiyear growing season. Our results showed that multiple planting durations decreased aboveground plant C and N concentrations and the C-N ratio (**Table 1**). These findings are supported by years of continuous planting, soil nutrient deficiency, gradual alfalfa grassland degradation, and plant biomass decrease due to self-toxic and self-thinning effects, which eventually results in less photosynthetic function and a lack of C assimilates in alfalfa grasslands (Zhang et al., 2017). At the same time, the results of **Table 1** indicated that the N concentration of alfalfa roots increased with increasing planting years, which is similar to the findings of other studies (Cao et al., 2020). The higher N concentrations of roots in plantations than those in natural-restored grassland can be explained by the N_2_ fixation of leguminous forage (Song et al., 2021). Additionally, in our paper, we found that alfalfa planting duration resulted in soil C and N decreases in the topsoil layer compared to the control (**Figure 2**). These results were mainly attributed to decreased plant inputs due to yearly plant removal, a decrease in outside litter input, and increased decomposition rates (Zhang J. H. et al., 2015). Moreover, the degree of C and N decrease depends on many factors, such as basic soil SOC and TN contents, climate conditions, and surface erosion status (Yang et al., 2019).

The SOC content of soil aggregates is a microscopic expression of SOC balance and mineralization rate, which is of great significance to soil fertility and soil carbon sequestration, and the TN content of soil is one of the main indicators of soil fertility (Lu et al., 2021). In this experiment, alfalfa grassland planted for 5 years had higher SOC and TN contents, and the largest SOC and TN storage were found in particles >2 mm and <0.25 mm, whereas the lowest SOC and TN contents were found in particles of 0.25–2.00 mm (**Figure 3**). This is mainly because macroaggregates are made up of many microaggregates and because of the formation of microaggregates through the combination of organic molecules with clay and cations and not by aggregated particles (Devine et al., 2014). Therefore, soil macroaggregates and microaggregate are rich in organic matter, and soil macroaggregates have a faster turnover time than middle aggregates (Puget et al., 2000). The microaggregates and the surrounding small particles combine to form large aggregates; when the large soil aggregates decompose into microaggregates, the particulate organic matter decomposes, resulting in a greatly reduced SOC content of the microaggregates (Bissonnais and Arrouays, 1997). At the same time, the binding and bonding effects of macroaggregates also reduces the SOC of microaggregates (Tisdall and Oades, 1982). Previous studies have shown that the SOC content of aggregates mainly exists in the particle size of <0.25 mm, which is a combination of smaller organic and inorganic colloids. The smaller aggregates have a larger specific surface area after being combined, resulting in more adsorbed organic matter (He et al., 2011).

Considering the aggregate size distribution and the SOC and TN of specific aggregates, the aggregate size distribution not only represents the contribution rate of aggregates to SOC and TN, but it might also completely depict the interaction of different planting durations to the soil C and N pools (Xu et al., 2012). In different numbers of alfalfa planting years, the stocks of SOC and TN in different proportions of aggregates were mainly restricted by aggregates > 2 mm, which were significantly higher than those of aggregates of other sizes (**Figure 4**). After 5 years of planting, the SOC and TN reserves of the soil aggregates were the highest. This is because, after 5 years of planting, the soil formed a stable plant community, increased the biomass of underground roots, and accumulated more SOC in the soil. At the same time, alfalfa is a leguminous crop with strong nitrogen fixation ability. The roots fix atmospheric nitrogen, which is conducive to the accumulation of soil nitrogen (Liu et al., 2005; Thomas et al., 2009). In our study, the soil C and N stocks decreased after alfalfa planting duration, which may be caused by the very low C and N inputs from plants and soil. Aminiyan et al. (2015) reported higher aggregate-related SOC content in larger aggregate fractions (> 2 mm). In short, large aggregates > 2 mm are the main contributors to the SOC and TN contents in soil aggregates. This may be due to the increase in the strength or stability of the aggregates due to the humidification of crop residues, thereby increasing the SOC concentration of the large aggregates (Kushwaha et al., 2001). Therefore, increasing the number of aggregates with a particle size greater than 2 mm can enhance soil carbon and nitrogen fixation. These findings may be limited by the method of observing spatial and temporal changes used in this article. However, we will focus on research on long-term positioning to overcome these shortcomings in the future.

### 4.4 The degradation mechanism of perennial alfalfa grasslands

Obtaining sustainable high biomass and suitable soil nutrient management is crucial for cultivate alfalfa grassland (Fang et al., 2021); however, degraded continuous alfalfa grasslands are frequently characterized by substantially reduced of biomass output and potential losses in soil nutrient (Gu et al., 2018). In the Loess Plateau of China, the low precipitation and high evapotranspiration of alfalfa field will limit its sustainable development (Ren et al., 2010). The lack of soil water content is the mainly parameter caused the perennial alfalfa grassland degradation (Wang et al., 2019). Alfalfa is a deep root plant that gives it access to water deeper in the soil than annual pastures and crops (Cheng et al., 2005). Alfalfa used soil water from deeper soil profile than other crops and extracted more water and thus creates a large soil water deficit (Huang et al., 2018). Previous study showed that the length of the alfalfa cropping phase in the short term (2–4 years) depends on soil water replenishment, and alfalfa continuously, for 6 years, reduces a relatively desiccation layer in the soil with 2-m to 10-m depth (Li, 1983). Li and Chao (1992) also found that the biomass output of continuous alfalfa markedly decreased after 7 or 8 years due to the declined of soil water. Jia et al. (2009) reported that the alfalfa average yield reached a peak after continuous 9 years planting and, more than 11 years, cultivated had higher soil water use efficiency. In our results, we also found that the soil water content was quickly declined after continuous cultivated alfalfa for 8 years (**Figure 2A**), the dynamic of underground biomass (the underground biomass reached the higher value in 8 year) (**Table 1**), because the longer the growing years of alfalfa, the deeper its root distribution, which intensified the high water consumption at deep soil (there was no rainfall when we collected soil moisture samples during the first half of the month). On the contrary, the soil water content increased from 10 to 20 years; this was probably because the alfalfa plants’ low transpiration and low productivity made less water consumption. The above results were coincided with the found by Li and Huang (2008) and Ren et al. (2010) and Li et al. (2018). Therefore, the long alfalfa stand duration may deplete available soil water, which would negatively impact production (Li and Huang, 2008) and thus caused the alfalfa grassland degradation.

On the other hand, the deficiency of soil nutrient also limits the alfalfa grassland sustainable development, and eventually leading to alfalfa grassland degradation and, conversely, enhancing the fertilizer application in perennial alfalfa can boost biomass output and prolong the alfalfa degradation timeline (Hakl et al., 2016; Gu et al., 2018; Fang et al., 2021). The decrease of soil nutrients, especially soil nitrogen, limits the perennial alfalfa growth, which, in turn, becomes a crucial restricting factor for the alfalfa cultivation (Yuan, 2017; Fang et al., 2021). In our results, we found that the soil C concentration decreased in the eight year and then increased after the 10th year of alfalfa cultivation (**Figure 2**). The soil C dynamical dramatically declined mainly because the fertilizer was applied in early planting alfalfa, and the carbon source in the soil is increased. With the continuous cultivated, a large amount of aboveground biomass was removed from alfalfa grassland, and the less carbon source will add to soil. In addition, the root biomass litters were not converted to carbon source by microbe decomposition. However, after cultivation alfalfa for the 10th to 20th years, the soil C storage increased, especially in the microaggregate, and this agrees with the findings from other researchers (Li and Huang, 2008). It seems that soil C storage rose through enhancing the aggregate stability with the increased in cultivation years. However, in this study, TN content continues decreased compared to the fifth year of alfalfa cultivation (**Figure 2**), mainly because the N that was derived from symbiotic fixation by perennial alfalfa over 20th years does not reach the N value of farmland, and artificial nitrogenous fertilizer losses were larger than N fixation by perennial alfalfa. However, the TN storage in the microaggregate presented less declined and finally increased after continuous cultivation on the 15th to 20th years (**Figure 4**). This was probably because this growing stage reached an imbalance between TN fixation and loss and reduced the consumption of TN on account of decreased alfalfa plant. Similar results were reported by Ji et al. (2020), who found that the highest values of SOC and TN content were observed in the begin stage of alfalfa grassland, and, with the cultivated prolong, the above contents declined, and the decline soil nutrients restricted alfalfa growth and biomass output.

## 5 Conclusion

We first found the ecological indicator of C and N stoichiometry from plant to soil aggregate fractions affecting soil degradation. Alfalfa plantations can improve soil structure and promote the number of macroaggregates within a certain year. Different planting periods display different effects on the SOC and TN distribution of aggregates. In the present study, the inputs of organic C compounds via root exudates provide a material base for the rapid turnover of C and N stocks. Both low-quality resources (high C/N) and high-quality material supply (low C/N) exert their effects on sustaining soil nutrients. Thus, alfalfa production can promote larger aggregates and accordingly improve soil C and N in large aggregates. This process would help improve the soil structure under the condition of continuous alfalfa planting. However, after 5–8 years of planting, alfalfa grassland productivity and soil quality began to degrade gradually. In conclusion, a certain period of alfalfa planting strengthened the stability of the soil physical structure, yet it resulted in a steady deterioration of the C and N stocks from soil to plant. Our findings provide novel insight into perennial alfalfa grassland degradation and, therefore, help explore a potentially sustainable solution in semiarid rainfed agricultural areas.

## Data Availability

The original contributions presented in the study are included in the article/supplementary material. Further inquiries can be directed to the corresponding authors.
